# The effect of exercise interventions on Irisin level: a systematic review and meta-analysis of randomized controlled trials

**DOI:** 10.17179/excli2022-4703

**Published:** 2022-02-25

**Authors:** Gholam Rasul Mohammad Rahimi, Keyvan Hejazi, Martin Hofmeister

**Affiliations:** 1 Department of Sports Sciences, Vahdat Institute of Higher Education, Torbat-e-Jam, Iran; 2 Department of Physical Education and Sport Sciences, Hakim Sabzevari University, Sabzevar, Iran; 3 Department Food and Nutrition, Consumer Center of the German Federal State of Bavaria, Munich, Germany

**Keywords:** Irisin, exercise intervention, FNDC5, PGC-1alpha, meta-analysis

## Abstract

Irisin is a hormone that is offered to be a hopeful remedial target in obesity and type 2 diabetes. It has received striking attention recently, whereas, the interactions between exercise training and irisin are still unclear. Therefore, this systematic review and meta-analysis investigated the impacts of exercise interventions on circulating irisin in adults. A systematic search was conducted in PubMed, CINAHL, MEDLINE, Cochrane, Google Scholar, and Scopus up to July 15, 2021. Twenty-four studies, which assessed a total of 921 participants were included and analyzed using a random-effects model to estimate weighted mean differences (MD) with 95 % confidence intervals (CI). Overall, data revealed that exercise training significantly increased circulating irisin (MD: 0.01, 95 % CI: 0.00, 0.01, p = 0.005), and declined insulin (MD: -2.09, 95 % CI: -2.81, -1.37, p < 0.00001), glucose (MD: -12.89, 95 % CI: -16.52, -9.26, p < 0.00001), and insulin resistance (MD: -0.89, 95 % CI: -1.15, -0.62, p < 0.00001). Subgroup analysis revealed that irisin raised significantly when resistance training (p = 0.04) and combined training (p = 0.002) were applied, and for the type 2 diabetes and prediabetes (p = 0.002 for both) groups. Moreover, subgroup analysis by the type of intervention demonstrated that insulin reduced when aerobic training (p < 0.00001) and combined training (p = 0.0003) were employed, but glucose and HOMA-IR reduced after all three types of exercise training. These findings demonstrate that exercise interventions may produce ameliorations in circulating irisin. Further long-term studies are required to confirm these findings.

## Introduction

Physical activities are among the main factors for health development and prevention of various diseases. It can directly increase energy consumption or indirectly affect the regulation of energy reception and cost of energy through modifying the secretion of the involved hormones (Firth et al., 2020[26]). According to the available findings, exercise training causes morphological and metabolic adaptations such as increased mitochondrial biogenesis (Sanayei et al., 2021[68]) and increased total oxidation capacity in skeletal muscles (Fritzen et al., 2020[28]).

One of the main properties of physical exercises is transforming white adipose tissue into brown adipose tissue (Verduci et al., 2021[76]). As an endocrine organ, skeletal muscles could release myokines. Numerous studies have shown that exercise training up-regulates the expression of peroxisome proliferator-activated receptor-γ coactivator (PGC-1α), demonstrating the potential to activate some genes produced in muscles, such as irisin protein (coded by fibronectin type III domain containing 5 (FNDC5) present in skeletal muscles (Boström et al., 2012[11]). Enhanced expression of FNDC5 in mice leads to a 3-4-fold increase of irisin, which is due to the subcutaneous brown adipose tissue and heat production (Boström et al., 2012[11]; Haas et al., 2012[30]). As a bridge between skeletal muscle and other tissues involved in homeostasis and energy metabolism, including the fatty tissue, irisin is induced by PGC-1α (Boström et al., 2012[11]). Irisin production causes the browning of human and mouse white adipose tissues (Boström et al., 2012[11]), leading to the expression of uncoupling protein 1 (UCP1) (Vliora et al., 2022[77]) and thus, result in enhanced oxidation of fatty acids and heat production. In other words, irisin induces raised heat and energy expenditure, glucose homeostasis, and eventually weight reduction (Boström et al., 2012[11]). PGC-1α is a PPAR-γ transcription factor activator that exerts the major part of its biological effects on energy metabolism (Hosseini et al., 2021[35]). Moreover, it has been illustrated that irisin may mediate some beneficial impacts of exercise in humans, such as mitochondrial biogenesis, angiogenesis, muscle fiber shifting, and preventing muscular atrophy (Jodeiri Farshbaf and Alviña, 2021[41]).

It has been suggested that brown adipose tissue can be utilized as a potential agent for diabetes management, as it ameliorates blood glucose control (Lapa et al., 2017[47]). In this context, experimental data reveal that decreased irisin levels might be associated with increased insulin resistance and glucose tolerance. Clinical studies have also demonstrated a decrease in FNDC5 expression in muscle and subcutaneous adipose tissue (Choi et al., 2013[15]). Furthermore, various studies have reported a lower serum level of irisin in type 2 diabetes patients versus healthy individuals (Choi et al., 2013[15]; Zhang et al., 2016[79]). Therefore, irisin may have utility as a preventive agent to tackle obesity and metabolic diseases (Arhire et al., 2019[4]).

To date, little is known about the type of exercise training needed to produce phenotypic changes in adipose tissue to improve health. Although recent studies have shown that the increased irisin levels due to physical exercise are associated with signs of decreased metabolic syndrome and insulin resistance (Elizondo-Montemayor et al., 2018[23]), there are contradicting results concerning the outcomes of exercise interventions on the conversion of white adipose tissue to brown adipose tissue and their role in the prevention of obesity. For instance, a 2015 systematic review by Qiu et al. (2015[63]) studied the impacts of long-term exercise on circulating irisin in adults; the results revealed that chronic resistance training had a moderate and significant effect in reducing irisin compared with the control group, while endurance exercise only had a trend toward significance. By contrast, a 2018 systematic review by Fox et al. (2018[27]) investigated the effect of an acute bout of exercise on the magnitude of post-exercise circulating irisin in adults; the authors concluded that irisin concentration increases substantially immediately after an acute bout of exercise. Furthermore, the recent systematic review by Cosio et al. (2021[16]) examined the efficacy of chronic resistance training on circulating irisin in adults. Data suggested that resistance training regimens seemed to elevate circulating irisin, especially in older adults and in demanding and progressive training interventions.

Although many studies are being conducted on irisin, the physiological functions of this myokine in humans and the effects of various exercise training on its expression are still ambiguous (Nygaard et al., 2015[57]). In addition, the limited number of high-quality trials directly investigating different modes of physical exercise regimens prevents any judgments on the most efficient influence on irisin. As a result, before recommending exercise programs as a non-pharmacological therapeutic measure and providing a final opinion on its positive impacts, the need for additional investigations seems necessary (Norheim et al., 2014[56]; Oelmann et al., 2016[58]).

Four meta-analyses on exercise training efficacy on irisin levels were conducted previously (Cosio et al., 2021[16]; Jandova et al., 2021[40]; Motahari Rad et al., 2021[53]; Qiu et al., 2015[63]); nevertheless, their participants' range of age (Jandova et al., 2021[40]) and level of physical activity (Motahari Rad et al., 2021[53]; Qiu et al., 2015[63]), and type of intervention (Cosio et al., 2021[16]) differed from the current meta-analysis. Moreover, both non-randomized and randomized controlled trials (RCTs) were included in these reviews (Jandova et al., 2021[40]; Qiu et al., 2015[63]). Therefore, the current review aimed to synthesize the pooled data from RCTs and controlled trials to examine the efficacy of exercise training regimens for changing circulating irisin and insulin resistance in adults.

## Methods

### Search strategy

A detailed search was performed using five databases, including PubMed, CINAHL, and Medline, Google Scholar, and Scopus. Searches included a mix of medical subject headings and free-text terms associating with the keywords "irisin", "FNDC5", "exercise training", "resistance training", "aerobic training", "strength training", "walking", "combined aerobic and resistance training", "circuit training", "circuit weight training", "interval training", "chronic exercise", "myokines", and "physical activity". The Boolean search terms (AND, OR, or NOT) were used, we merged the search terms; exercise training participation with the term irisin and/or FNDC5. The search strategy covered the period from database inception until July 15, 2021. Then, following the initial screening, systematic reviews, meta-analyses, and all study references were also searched to ensure that all relevant articles established on a basis on the inclusion and exclusion criteria. The current study adhered to the Preferred Reporting Items for Systematic Reviews and Meta-Analyses (PRISMA) guidelines (Page et al., 2021[60]).

### Selection criteria

The participants, intervention, comparison, outcome, time, and study design (PICOTS) criteria were considered to determine the study inclusion criteria. The titles, abstracts, and full text were independently reviewed by two investigators to appropriate articles to establish study eligibility. Exercise training RCTs and controlled trials in adults were included. In the current meta-analyses, exercise interventions comprised aerobic, resistance, and combined (aerobic + resistance) training. Studies included in this review compared adults in the intervention and control groups.

### Inclusion/exclusion criteria

To study identification and selection, we used the criteria as follows: 1) full-text RCTs or control trials published in the English language; 2) adults (aged ≥ 18 years); 3) investigations that used aerobic, resistance, or combined aerobic and resistance training, in a pre-post design with a non-exercise control group; 4) studies reported circulating (plasma/serum) irisin levels. Review articles, literature reviews, conferences, abstracts, and study protocols have been omitted.

### Outcome measures

The primary outcome was plasma/serum irisin level (in µg/mL), which was measured using a standardized commercial Enzyme-Linked Immunosorbent Assay (ELISA) kit. The secondary outcome measures were fasting glucose (in mg/dl), insulin (in mcUI/mL), and HOMA-IR.

### Data extraction

Two authors (GRMR and KH) individually extracted the data, and disagreements between us were resolved by discussion. The information extracted comprised author, year of publication and country, the number of cases and controls, mean age of participants, health status, features of the exercise programs, mean and standard deviation (SD) of the outcome measures at baseline, post-intervention and/or changes between baseline and post-intervention.

### Data synthesis

The effect size of any outcomes was summarized by calculating the mean difference (MD) between the exercise intervention and control condition from pre-intervention and post-intervention for all included papers. We evaluated and detailed independently any result if each research reported different results for the current study. Given similar reporting techniques for outcomes, the mean difference (MD) with 95 % confidence interval (CI) was applied. Review Manager 5.3 (The Nordic Cochrane Centre, Copenhagen, Denmark) was employed for performing all analyses. Extracted outcome data were completed by the change in the mean and SD values.

The mean at baseline was subtracted from the post-intervention mean, and the change SD was computed using for study group participant numbers in conjunction with group p-values or 95 % CI where the change in mean and SD was not reported. In articles that reported standard error of the mean (SEM) data instead of the SD, this value was converted to SD (Higgins et al., 2003[34]). Where data were not presented in text or tables, and authors could not be contacted, data revealed in figures were extracted or obtained where feasible by GetData Graph Digitizer software. Where an article contained a control group and more than one exercise group, we separately labeled each exercise group and divided the sample size of the control group by the number of exercise groups.

Pooled estimates of the effect of exercise on outcome measures were obtained using a random-effects model. The heterogeneity among the studies was evaluated by the I2 statistic, with values >50 % considered to show substantial heterogeneity (Higgins et al., 2003[34]). Subgroup analyses were employed to recognize potential causes of heterogeneity among the studies. Exercise training modality (aerobic, resistance, combined aerobic and resistance), health status (e.g., patients with multiple sclerosis, type 2 diabetes, and metabolic syndrome), body mass index classification (BMI < 25 kg/m^2^, or ≥ 25 kg/m^2^), and gender were considered as the predefined sources of heterogeneity. Meta-analysis was carried out using Forest plots and used a 5 % level of significance to reveal the significance of results. The risk of publication bias was assessed employing Funnel plots (Egger et al., 1997[22]), considering p < 0.05 a statistically significant bias.

### Study quality 

The methodological quality of the investigations was examined independently based on a fifteen-point scale, Tool for the Assessment of Study Quality and Reporting in Exercise (TESTEX), which is a validated tool for evaluating the quality (5 points maximum) and reporting (10 points maximum) of exercise training studies (Smart et al., 2015[71]). Two independent reviewers (GhRMR and KH) conducted this evaluation, and any disagreements between us were resolved by consensus.

## Results

### Study and participant characteristics

**Figure 1:**
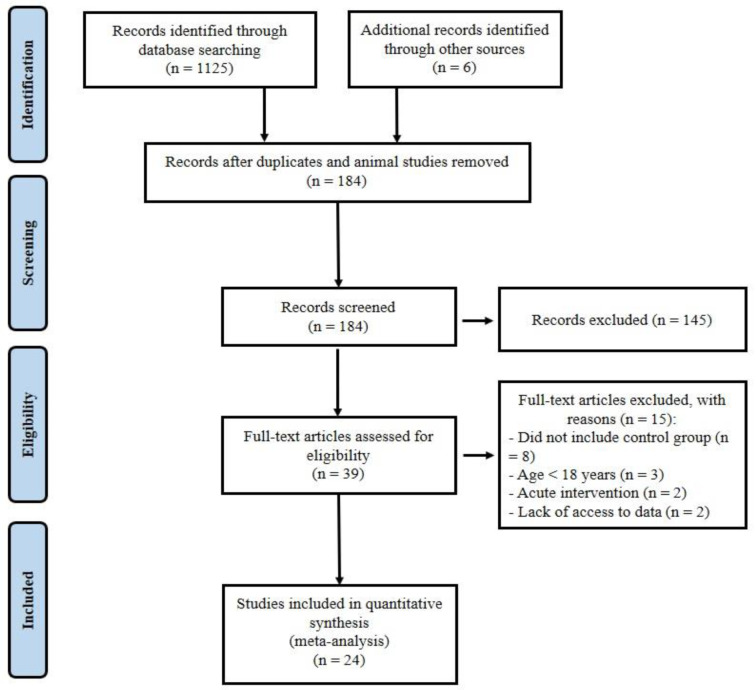
PRISMA flow diagram

One thousand one hundred and thirty-one records were identified in the initial search. After removing duplicates and animal researches (n = 947), the remaining papers were screened based on the title and the abstract, and then 145 articles were omitted, leaving 39 full-text articles. Fifteen other articles were removed because of the following reasons: (a) did not include control group (Brinkmann et al., 2022[13]; Dünnwald et al., 2019[21]; Norheim et al., 2014[56]; Otero-Díaz et al., 2018[59]; Rashid et al., 2020[64]; Sari-Sarraf et al., 2017[69]; Tsuchiya et al., 2015[75]; Winn et al., 2017[78]), (b) participants with mean age <18 years (Archundia-Herrera et al., 2017[3]; Blizzard LeBlanc et al., 2017[9]; Dundar et al., 2021[20]), (c) acute trials (a training period less than two weeks or performing only an acute exercise testing) (Kraemer et al., 2014[45]; Tsuchiya et al., 2014[74]), (d) lack of access to data (Hecksteden et al., 2013[32]; Micielska et al., 2019[49]). Twenty-four articles (Amanat et al., 2020[2]; Azimi Rashti et al., 2019[6]; Bagheri et al., 2020[7]; Banitalebi et al., 2019[8]; Bonfante et al., 2017[10]; Briken et al., 2016[12]; Dianatinasab et al., 2020[19]; Enteshary et al., 2019[24]; Ghanbari-Niaki et al., 2018[29]; Jafari et al., 2020[37]; Jaffari et al., 2020[38]; Kim et al., 2016[42]; Korkmaz et al., 2019[44]; Miyamoto-Mikami et al., 2015[50]; Motahari Rad et al., 2020[52]; Murawska-Cialowicz et al., 2020[54]; Nazari et al., 2017[55]; Pekkala et al., 2013[61]; Poutafkand et al., 2020[62]; Rezaeimanesh, 2020[65]; Safarimosavi et al., 2021[67]; Scharhag-Rosenberger et al., 2014[70]; Tofighi et al., 2017[73]; Zhao et al., 2017[80]) met our inclusion criteria and were entered in the meta-analysis (PRISMA flow diagram; Figure 1(Fig. 1)). Studies were carried out in Iran (15), Germany (2), Finland (2), Japan (1), Poland (1), South Korea (1), China (1), and Brazil (1). The 24 included articles had 921 subjects, 590 (64 %) subjects in the intervention group, and 331 (36 %) in the control group.

### Intervention details

The relevant characteristics for each of the included studies are provided in Supplementary Table 1. The intervention period of studies varied from 8 to 24 weeks, with each session's length of range 15-90 min. Twelve studies exclusively recruited male subjects (Bagheri et al., 2020[7]; Bonfante et al., 2017[10]; Jafari et al., 2020[37]; Jaffari et al., 2020[38]; Korkmaz et al., 2019[44]; Motahari Rad et al., 2020[52]; Murawska-Cialowicz et al., 2020[54]; Nazari et al., 2017[55]; Pekkala et al., 2013[61]; Rezaeimanesh, 2020[65]; Safarimosavi et al., 2021[67]; Zhao et al., 2017[80]), eight studies exclusively recruited female subjects (Amanat et al., 2020[2]; Azimi Rashti et al., 2019[6]; Banitalebi et al., 2019[8]; Dianatinasab et al., 2020[19]; Enteshary et al., 2019[24]; Ghanbari-Niaki et al., 2018[29]; Poutafkand et al., 2020[62]; Tofighi et al., 2017[73]), and four studies recruited both males and females (Briken et al., 2016[12]; Kim et al., 2016[42]; Miyamoto-Mikami et al., 2015[50]; Scharhag-Rosenberger et al., 2014[70]). In 22 studies, the mean age of subjects varied from 22.5 to 62.1 years. Two studies (Miyamoto-Mikami et al., 2015[50]; Rezaeimanesh, 2020[65]) did not report the mean age of participants. Based on BMI classification criteria, four investigations had subjects who were classified on average as obese, 14 as overweight, four as the normal weight, with two not reporting mean BMI (Briken et al., 2016[12]; Enteshary et al., 2019[24]). Studies specifically recruited participants with type 2 diabetes (three investigations), metabolic syndrome (two investigations), obese-only subjects (five investigations), overweight subjects (seven investigations), or both obese and overweight (two investigations). One investigation recruited exclusively for prediabetes and one for multiple sclerosis.

Three studies employed aerobic exercise training, four studies investigated resistance training, four studies examined isolated aerobic and resistance training, two studies used all three modalities (aerobic, resistance, and combined aerobic + resistance training), three studies employed high-intensity interval training, and two studies examined isolated high-intensity interval training and continuous endurance training. Moreover, one study used isolated aerobic and combined aerobic + resistance training, two studies reviewed combined aerobic + resistance training, one study employed isolated high-intensity interval training and moderate-intensity interval training, one study used isolated sprint interval training and combined aerobic + resistance training and one study employed isolated resistance training + high-intensity interval training and resistance training + moderate-intensity continuous training.

### Meta-analysis results

#### Change in irisin

**Figure 2:**
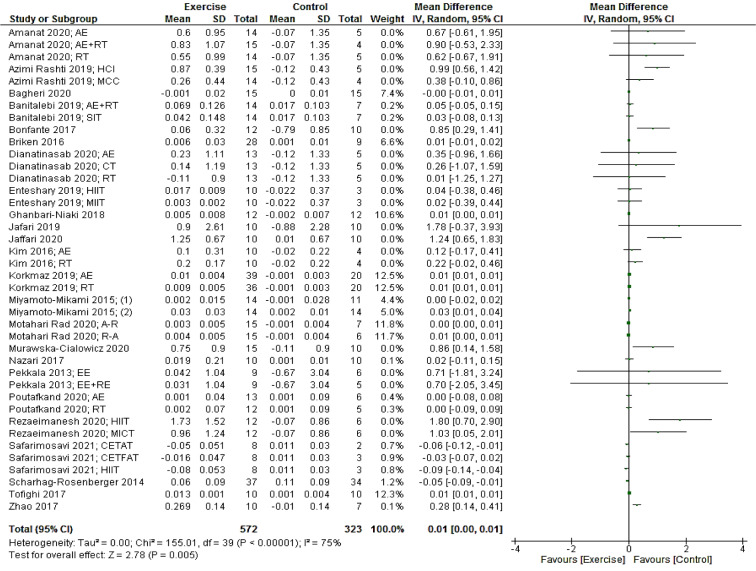
Forest plot of the effects of exercise training versus control on irisin. AE, aerobic; RT, resistance training; HCI, high-intensity concurrent interval; MCC, moderate-intensity concurrent continues; SIT, sprint interval training; HIIT. High intensity interval training; A-R, aerobic-resistance; R-A, resistance-aerobic; EE, endurance exercise; CETFAT, continuous endurance training with intensity equivalent to Fatmax (maximal fat oxidation); CETAT, continuous endurance training with intensity equivalent to anaerobic threshold

Based on 40 intervention arms, exercise interventions significantly increased irisin [0.01 µg/mL (95 % CI, 0.00 to 0.01), p = 0.005] when compared with a control group (Figure 2(Fig. 2); References in Figure 2: Amanat, 2020[2]; Azimi Rashti, 2019[6]; Bagheri 2020[7]; Banitalebi, 2019[8]; Bonfante, 2017[10]; Briken, 2016[12]; Dianatinasab, 2020[19]; Enteshary, 2019[24]; Ghanbari-Niaki, 2018[29]; Jafari, 2019[37]; Jaffari, 2020[38]; Kim, 2016[42]; Korkmaz 2019[44]; Miyamoto-Mikami, 2015[50]; Motahari Rad, 2020[52]; Murawska-Cialowicz 2020[54]; Nazari, 2017[55]; Pekkala, 2013[61]; Poutafkand, 2020[62]; Rezaeimanesh, 2020[65]; Safarimosavi, 2021[67]; Scharhag-Rosenberger, 2014[70]; Tofighi, 2017[73]; Zhao 2017[80]). There was a significant moderate heterogeneity (I^2^ = 75 %, p < 0.00001) among studies included for this comparison. 

Subgroup analysis by exercise training modalities revealed a significant increase in irisin for studies with resistance [0.01 µg/mL (95 % CI, 0.00 to 0.02), p = 0.04; I^2^ = 79 %, p < 0.00001; 22 interventions] and combined aerobic + resistance [0.00 µg/mL (95 % CI, 0.00 to 0.01), p = 0.002; I^2^ = 0 %, p = 0.72; six interventions] protocols, but not for studies with aerobic training protocols [-0.01 µg/mL (95 % CI, -0.03 to 0.02), p = 0.60; I^2^ = 70 %, p = 0.0001; 12 interventions]. 

Subgroup analyses by health status of participants revealed a significant change in irisin for patients with type 2 diabetes [0.00 (95 % CI, 0.00 to 0.01), p = 0.002; I^2^ = 0.0 %; six interventions] and prediabetes [-0.06 (95 % CI, -0.10 to -0.02), p = 0.002. I^2^ = 40 %; three interventions]. In investigations that included metabolic syndrome populations, however, there was not a significant change reported for irisin [0.45 (95 % CI, -0.08 to 0.99), p = 0.10; I^2^ = 0.0 %; six interventions]. 

In addition, subgroup analyses by BMI and gender did not reveal any significant effects (see Supplementary Table 2).

#### Change in fasting insulin, fasting glucose, and insulin resistance

**Figure 3:**
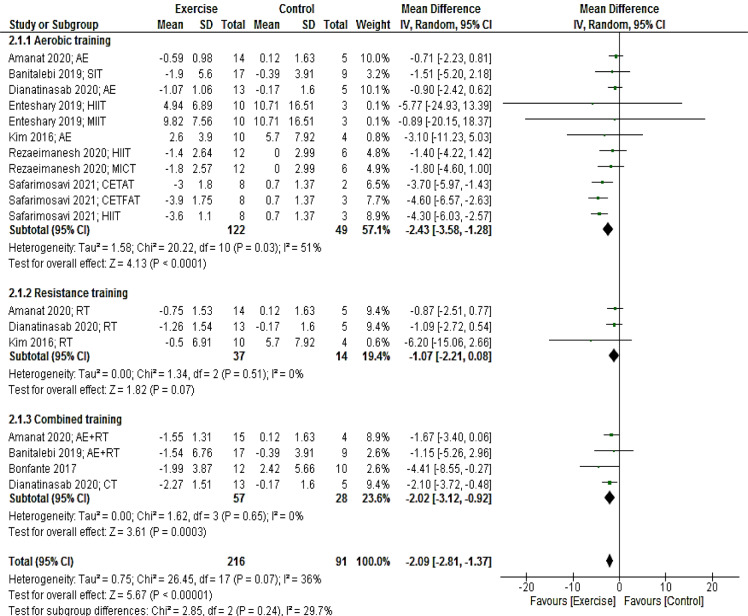
Forest plot of the effects of exercise training versus control on insulin. AE, aerobic; RT, resistance training; SIT, sprint interval training; HIIT. High intensity interval training; CETFAT, continuous endurance training with intensity equivalent to Fatmax (maximal fat oxidation); CETAT, continuous endurance training with intensity equivalent to anaerobic threshold

Eighteen arms providing a total of 307 participants reported fasting insulin as an outcome measure. Pooled results from the random-effects model revealed that exercise training significantly decreased insulin levels (MD: -2.09 mcUI/mL; 95 % CI [-2.81, -1.37]; p < 0.00001). When we stratified studies based on the mode of exercise training (aerobic, resistance, and combined), we found a significant reduction in insulin after aerobic (MD: -2.43 mcUI/mL; 95 % CI [-3.58, -1.28]; p < 0.0001; 11 interventions) and combined (MD: -2.02 mcUI/mL; 95 % CI [-3.12, -0.92]; p = 0.0003; four interventions) training, but not after resistance training (MD: -1.07 mcUI/mL; 95 % CI [-2.21, 0.08]; p = 0.07; three interventions) (Figure 3(Fig. 3); References in Figure 3: Amanat, 2020[2]; Banitalebi, 2019[8]; Bonfante, 2017[10]; Dianatinasab, 2020[19]; Enteshary, 2019[24]; Kim, 2016[42]; Rezaeimanesh, 2020[65]; Safarimosavi, 2021[67]). 

**Figure 4:**
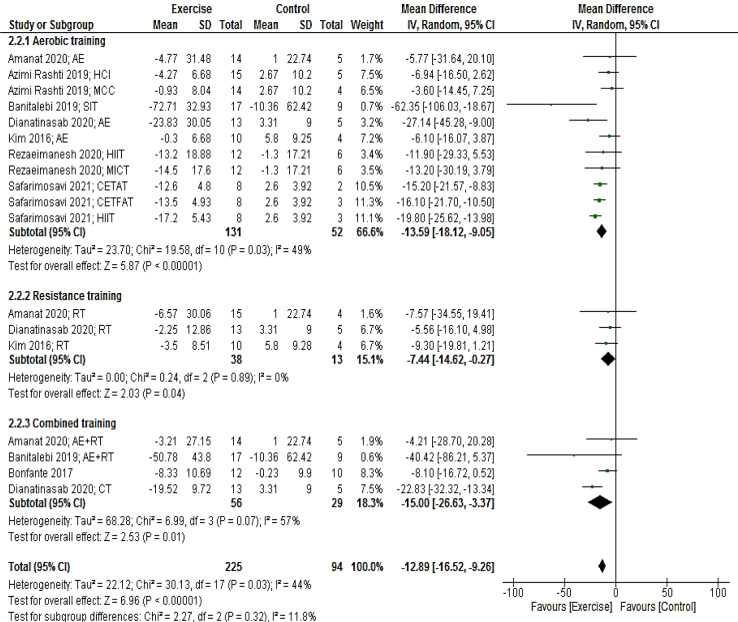
Forest plot of the effects of exercise training versus control on glucose. AE, aerobic; RT, resistance training; SIT, sprint interval training; HIIT. High intensity interval training; CETFAT, continuous endurance training with intensity equivalent to Fatmax (maximal fat oxidation); CETAT, continuous endurance training with intensity equivalent to anaerobic threshold

Eight studies (18 arms) presenting a total of 319 participants reported fasting glucose as an outcome measure. Pooled results showed that glucose levels reduced in the intervention group compared with the control group (MD: -12.89 mg/dL; 95 % CI [-16.52, -9.26]; p < 0.00001). Subgroup analyses by the mode of exercise training revealed a significant reduction for studies with aerobic (MD: -13.59 mg/dL; 95 % CI [-18.12, -9.05]; p < 0.00001; 11 arms), resistance (MD: -7.44 mg/dL; 95 % CI [-14.62, -0.27]; p = 0.04; three arms), and combined (MD: -15.00 mg/dL; 95 % CI [-26.6, -3.37]; p = 0.01; four arms) training (Figure 4(Fig. 4); References in Figure 4: Amanat, 2020[2]; Azimi Rashti, 2019[6]; Banitalebi, 2019[8]; Bonfante, 2017[10]; Dianatinasab, 2020[19]; Kim, 2016;[42] Rezaeimanesh, 2020[65]; Safarimosavi, 2021[67]).

Eight studies (18 arms) providing a total of 324 participants reported HOMA-IR as an outcome measure. Pooled results presented that HOMA-IR reduced in the intervention group compared with the control group (MD: -0.89; 95 % CI [-1.15, -0.62]; p < 0.00001). 

**Figure 5:**
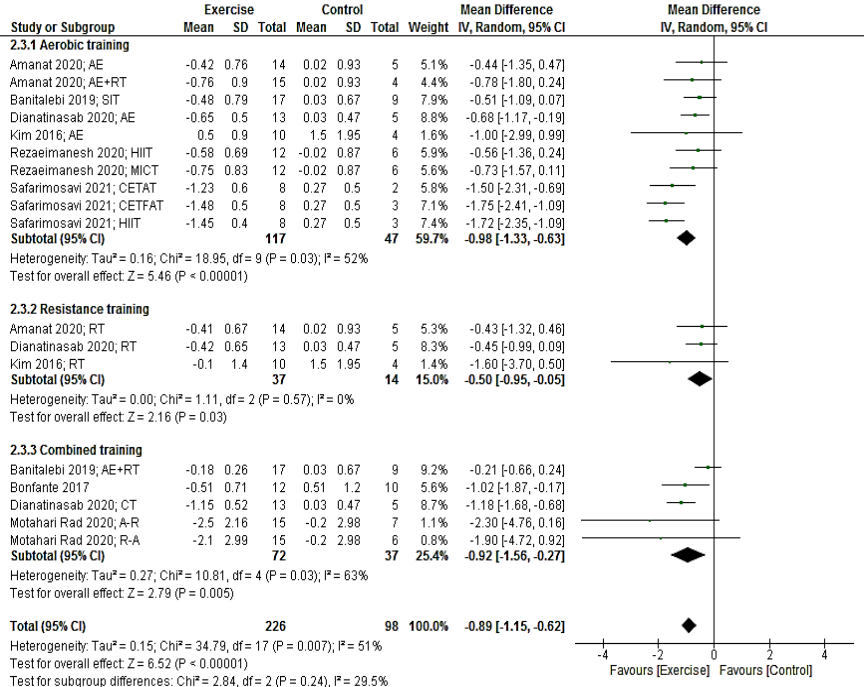
Forest plot of the effects of exercise training versus control on HOMA-IR. AE, aerobic; RT, resistance training; SIT, sprint interval training; HIIT. High intensity interval training; CETFAT, continuous endurance training with intensity equivalent to Fatmax (maximal fat oxidation); CETAT, continuous endurance training with intensity equivalent to anaerobic threshold

Subgroup analyses by the mode of exercise training revealed a significant reduction for studies with aerobic (MD: -0.98; 95 % CI [-1.33, -0.63]; p < 0.00001; 10 arms), resistance (MD: -50; 95 % CI [-0.95, -0.05]; p = 0.03; three arms) and combined (MD: -0.92; 95 % CI [-1.56, -0.27]; p = 0.005; five arms) training (Figure 5(Fig. 5); References in Figure 5: Amanat, 2020[2]; Banitalebi, 2019[8]; Bonfante, 2017[10]; Dianatinasab, 2020[19]; Kim, 2016[42]; Motahari Rad, 2020[52], Rezaeimanesh, 2020[65]; Safarimosavi, 2021[67]).

### Study quality

The overall methodological quality of included studies was estimated to be moderate to good, with a median TESTEX score of 9 (range 7-12) out of a maximum of 15. Two investigations scored 12, one study scored 11, five experiments scored 10, seven experiments scored 9, six experiments scored eight and three experiments scored 7 (see Supplementary Table 3). Of the TESTEX criteria, the following was done particularly poorly: physical activity monitoring in the control groups 0/24; intention to treat analyses only 1/24 study; allocation concealment only 4/24 studies; randomization specified 9/24 studies. The other criteria were each met in at least 50 % of investigations.

### Heterogeneity and publication bias

Our analyses demonstrated high heterogeneity in irisin (I^2^ = 75 %, p < 0.00001) and moderate heterogeneity in HOMA-IR (I^2^ = 51 %, p = 0.007), fasting glucose (I^2^ = 44 %, p = 0.03), and fasting insulin (I^2^ = 36 %, p = 0.07). Egger plots exhibited little to moderate evidence of publication bias as the standard error/mean difference plots were tightly grouped together (see Supplementary Figures 1-4).

## Discussion

The current meta-analysis included published randomized controlled trials (RCTs) investigating the efficacy of exercise training in circulating irisin and insulin resistance in adults. Based on 24 studies comprised of 40 intervention arms, exercise intervention significantly increased irisin compared with a control condition. Subgroup analysis by exercise training modalities revealed a significant increase in irisin for studies with resistance and combined aerobic + resistance protocols, but not for studies with aerobic training. Moreover, subgroup analyses by the health status of participants revealed a significant change in irisin for patients with type 2 diabetes and prediabetes. Investigations included metabolic syndrome populations, however, did not show a substantial alteration in irisin.

As a hormone derived from FNDC5, irisin is recognized as the target gene for PGC1-α. mRNA FNDC5 is expressed higher in muscles than in other organs (Huh et al., 2012[36]), and the increased expression of FNDC5 in skeletal muscles is significantly linked to the serum level of irisin (Huh et al., 2012[36]; Roca-Rivada et al., 2013[66]). Studies have displayed a robust association between muscle levels of mRNA FNDC5 and FNDC5 (Lecker et al., 2012[48]; Norheim et al., 2014[56]), supporting the role of PGC1-α as a regulator for FNDC5 and irisin. However, perhaps the isolated resistance and mixed aerobic + resistance interventions have induced the activation of effective signaling on the up-regulation of PGC1-α (Suwa et al., 2008[72]), resulting in FNDC5 expression and then elevation in circulating irisin. It has been reported that there is a correlation between changes in irisin concentration and lactate, both increments following physical activities, and it is suggested that the increase in irisin is due to muscle needs. A hypothesis states that muscle need might play a role in irisin's physiological regulation (Daskalopoulou et al., 2014[18]); the rise in irisin after acute exercise is associated with diminished muscular ATP level. There is the possibility that diminished ATP or increased ADP level in addition to phosphate group might activate irisin signaling to help store ATP homeostasis in muscular function (Huh et al., 2012[36]). Irisin level is correlated with the amount of ATP in the first place and with metabolites related to glycolysis and lipolysis in the skeletal muscle in the second place (Huh et al., 2012[36]). Engagement in exercise training leads to a further reduction in muscles' creatine phosphate, ATP, and glycolysis (Hargreaves et al., 1998[31]). While these exercises increase the activity of AMP-activated protein kinase, which per se activates PGC1-α and phosphorylation (Jäger et al., 2007[39]; Knutti et al., 2001[43]). In the present study, the reduction in ATP and increased activation of PGC1-α due to exercise training probably leads to the release of irisin from its primary source, skeletal muscle FNDC5, into the bloodstream. Part of these differences between various types and intensities of exercises are probably because of intracellular energy levels. In fact, it has been shown that conducting workouts at high intensity leads to increased AMP, ADP, AMP/ATP ratio, and lactate versus workouts at low intensity (Chen et al., 2003[14]). Moreover, the relationship between irisin and intracellular metabolites (Huh et al., 2012[36]), may partially explain the difference between various types of physical exercises on its level. Physiologically, it is unclear if raised serum level of FNDC5-derived irisin is in response to exercise training, or FNDC5 is stored before translation, or its raised serum level is because of increased muscle need. Alternatively, physical exercises might activate the breakdown of specific factors derived from FNDC5, leading to the rapid release of irisin into the bloodstream at the beginning of activity (Daskalopoulou et al., 2014[18]). The study by Norheim et al. (2014[56]) stated that the plasma level of irisin is probably independent of an increase in FNDC5. Nevertheless, further research is required to confirm the mechanisms underlying the impact of exercise training on irisin.

Our results illustrate that insulin, glucose, and insulin resistance significantly reduced after exercise interventions. When we stratified studies based on the mode of exercise training (aerobic, resistance, and combined), we found a significant reduction in insulin after aerobic and combined training, but not after resistance training. Subgroup analyses by the mode of exercise training revealed a considerable reduction of glucose and insulin resistance for studies with aerobic, resistance, and combined training. A number of recent meta-analyses reported that exercise training had a moderate-to-large impact on reducing glucose, insulin, and HOMA-IR in adults (Ashton et al., 2020[5]; Kumar et al., 2019[46]; Mohammad Rahimi et al., 2021[51]). Some mechanisms that could increase the function of insulin after aerobic exercises include increased insulin post-receptor signaling, increased expression of insulin-responding glucose transporter 4 (GULT4), increased activity of glycogen synthetase and hexokinase, decreased release and increased removal of free fatty acids, increased release of glucose from the blood into muscles due to increased muscular capillaries and the modified combination of muscles to increase glucose uptake (Eriksson et al., 1997[25]). Therefore, aerobic exercises are methods to decrease insulin resistance and the risk for developing type 2 diabetes, particularly in obese individuals. Insulin resistance may potentially be mediated by changes in the function of several peptide mediators secreted from adipocytes, including tumor necrosis factor-alpha (TNFα), leptin, and adiponectin (Alizaei Yousefabadi et al., 2021[1]). In non-inflammatory conditions, TNFα is derived from adipose tissue, and its plasma level is associated with body fatty tissue. TNFα interferes with insulin signaling by reducing signaling through serine phosphorylation. Adiponectin is secreted from adipocytes with an inverse proportion to adipocytes, and it is a potential TNFα inhibitor. The serum level of adiponectin decreases in obesity, insulin resistance, diabetes mellitus, and metabolic syndrome (Das, 2010[17]). Another reason for these contradicting findings could probably be due to differences in the duration, intensity, and exercise level of the subjects.

The strength of this meta-analysis is that we pooled all included studies in our analysis and compared the impact of various types of exercise training on circulating irisin and metabolic outcome measures; however, we acknowledge that there are some limitations to our findings. The existing sample size, similar to many other analyses on the effects of exercise training, was small, thereby restricting the generalizability of our results. Second, exercise intensity, duration, and period of the interventions varied substantially in the employed studies, which may have impacted our results. Third, in the main meta-analysis, there were small-study effects that could be attributed to the bias of publication, poor methodological quality, true heterogeneity, and chance (Higgins, 2011[33]).

## Conclusion

Our study displays that exercise training intervention may help improve irisin levels in adults, especially in type 2 diabetes and prediabetes patients. Moreover, isolated resistance exercise and combined aerobic and resistance exercise appear to be optimal in changes of irisin. Future studies are needed to clarify the mechanisms underlying the impact of exercise training on irisin in adults.

## Declaration

### Funding

This research did not receive any specific grant from funding agencies in the public, commercial, or not-for-profit sectors.

### Conflict of interest

The authors declare no potential conflicts of interest with respect to the research, authorship, and/or publication of this article.

## Supplementary Material

Supplementary information
