# Supplementary material for: The effect of exercise interventions on Irisin level: a systematic review and meta-analysis of randomized controlled trials
Source: EXCLI J. 2022 Feb 25;21:524–39. doi: 10.17179/excli2022-4703 (PMC9441678; doi:10.17179/excli2022-4703)
Supplement: Supplementary information [file EXCLI-21-524-s-001.pdf]

EXCLI Journal 2022;21:524-539 – ISSN 1611-2156
Received: January 18, 2022, accepted: February 15, 2022, published: February 25, 2022

Supplementary information to:

Review article:

THE EFFECT OF EXERCISE INTERVENTIONS ON IRISIN LEVEL: A SYSTEMATIC REVIEW AND META-ANALYSIS OF RANDOMIZED CONTROLLED TRIALS

Gholam Rasul Mohammad Rahimi[a], Keyvan Hejazi[b*], Martin Hofmeister[c]

[a] Department of Sports Sciences, Vahdat Institute of Higher Education, Torbat-e-Jam, Iran
[b] Department of Physical Education and Sport Sciences, Hakim Sabzevari University, Sabzevar, Iran
[c] Department Food and Nutrition, Consumer Center of the German Federal State of Bavaria, Munich, Germany

* **Corresponding author:** Keyvan Hejazi, Department of Physical Education and Sport Sciences, Hakim Sabzevari University, Sabzevar, Iran. Tel: +98 (51) 344012620, E-mail: k.hejazi@hsu.ac.ir

https://dx.doi.org/10.17179/excli2022-4703

This is an Open Access article distributed under the terms of the Creative Commons Attribution License (http://creativecommons.org/licenses/by/4.0/).

EXCLI Journal 2022;21:524-539 – ISSN 1611-2156
Received: January 18, 2022, accepted: February 15, 2022, published: February 25, 2022

Supplementary Table 1: Characteristics of the included studies

Author, year	Country	Disease	Participants				Modes of exercise	Intervention group
			EX (CON)	BMI	Age	Gender		
Amanat, 2020	Iran	MS	45 (15)	29.4	54.5	F	AE, RT, AE+RT	**AE group:**<br>3 sessions/week × 60 min, at 60-75 % HRmax; 12 weeks<br>**RT group:**<br>3 sessions/week; 3 sets × 10 reps at 60-80 % 1RM; 12 weeks<br>**AE+RT group:**<br>Participants performed both AE and RE simultaneously in one session. Each session was consisting of 20 min of walking on a treadmill, followed by 5 min rest and one set of RT, consisting of 10 different exercises similar to the RE exercise program. The intensities of the AE and RT exercises were gradually increased according to the AE and RE protocols, respectively.
Azimi Rashti, 2019	Iran	No disease	29 (9)	29.8	50.7	F	HCI, MCC	**HCI group (RT + HIIT):**<br>3 sessions/week × 50-65 min included 3-4 sets × 8-15 reps RT and 4 × 4 min intervals at 85-95 % HRmax followed by 4 min recovery at 56 % HRmax; 10 weeks<br>**MCC group (RT + CT):**<br>3 sessions/week × 50-65 min included 3-4 sets × 8-15 reps RT and continuous AE at 50-75 % HRmax
Bagheri 2020	Iran	Overweight	15 (15)	27.5	43.9	M	AE	3 sessions/week at 40–59 % HRR; 8 weeks
Banitalebi, 2019	Iran	T2D	34 (18)	28.9	55.6	F	SIT, AE+RT	**SIT group:**<br>3 sessions/week, 4×30 s, at 25-50 W/min; 10 weeks<br>**AE+RT group:**<br>3 sessions/week × 15-30 min, at 60–70 % MHR%; 10 weeks<br>3 sessions/week, 50 min, 1-3 sets × 10-15 reps; 10 weeks
Bonfante, 2017	Brazil	Obese	12 (10)	30.9	49.1	M	AE, RT	**RT group:**<br>3 sessions/week, 3 sets × 6-10 reps; 24 weeks<br>**AE group:**<br>3 sessions/week × 60 min, at 55–85 % VO_{2peak}; 24 weeks
Briken, 2016	Germany	Multiple sclerosis	32 (10)	-	50.2	Both	AE	2-3 sessions/week × 10-20 min, at 12.5-100 W/min; 9 weeks
Dianatinasab, 2020	Iran	MS	45 (15)	29.9	53	F	AE, RT, CT	**AE group:**<br>3 sessions/week × 30-60 min, at 60-75 % $\dot{V}O_{2max}$; 8 weeks<br>**RT group:**<br>2-3 sessions/week, 60 min, 2 sets × 8-10 reps at 60-80 % 1 Rmax; 8 weeks

EXCLI Journal 2022;21:524-539 – ISSN 1611-2156
Received: January 18, 2022, accepted: February 15, 2022, published: February 25, 2022

Author, year	Country	Disease	Participants				Modes of exercise	Intervention group
			EX (CON)	BMI	Age	Gender		
								**CT group:**<br>performed both AE and RT simultaneously in one session
Enteshary, 2019	Iran	T2D	16 (10)	-	40	F	HIIT, MIIT	**HIIT group**:<br>5 sessions/week × 30-45 min, at 60-70 % HRmax; 8 weeks<br>**MIIT group**:<br>5 sessions/week 55 % to 69 % HRmax; 8 weeks
Ghanbari-Niaki, 2018	Iran	Overweight	12 (12)	27.3	57.4	F	RT	3 sessions/week; 2 sets × 12 exercises at 55 % 1RM; 8 weeks
Jafari, 2019	Iran	Obese	10 (10)	28.9	29.4	M	HIT	3 sessions/ week × 45-60 min at intensity of 90 % HRR, 8 weeks
Jaffari, 2020	Iran	Obese	10 (10)	31.4	26.97	M	RT	3 sessions/week, 60 min, 3 sets × 6-14 reps at 55-75 % 1 Rmax; 8 weeks
Kim, 2016	South Korea	Overweight/Obese	20 (8)	26.6	26	Both	AE, RT	**AE group:**<br>5 sessions/ week × 60 min at intensity of 65-80 % HRM, 8 weeks<br>**RT group:**<br>5 sessions/ week × 60 min; 3 sets × 10-12 reps at 65-80 % 1RM, 8 weeks
Korkmaz 2019	Finland	Overweight/Obese	75 (40)	29.6	54.5	M	AE, RT	**AE group**:<br>3 sessions/week × 60 min at intensity of 55-75 % HRR, 12 weeks<br>**RT group:**<br>3 sessions/week × 60 min at intensity of 50-85 % 1RM, 12 weeks
Miyamoto-Mikami, 2015	Japan	No disease	28 (25)	22.5	-	Both	AE	3 sessions/week × 45 min, at 60-70 % $\dot{V}O_2$peak; 8 weeks
Motahari Rad, 2020	Iran	T2D	34 (17)	29.4	49.6	M	AE + RT	**AE group:**<br>3 sessions/week × 60 min, at 75-95 % HRmax; 12 weeks<br>**RT group:**<br>3 sessions/week; 3 sets/8-18 reps at 40-80 % 1RM; 12 weeks
Murawska-Cialowicz 2020	Poland	No disease	15 (10)	25.8	28.9	M	HIIT	2 sessions/week; 8 × 4 min intervals (8 × 20 s work with 10 s rest between each interval) followed by 1 min rest between each interval; 8 weeks
Nazari, 2017	Iran	Overweight	10 (10)	27	22.5	M	RT + AE	3 sessions/ week; 20 min at 50-85 % HRmax + 3 sets × 10 reps at 50-85 % 1RM, 8 weeks
Pekkala, 2013	Finland	No disease	18 (2)	25.8	59.5	M	EE, EE + RT	**EE group:**<br>3 sessions/ week; 60-90 min under the level of their aerobic threshold, between the aerobic-anaerobic thresholds, or above

EXCLI Journal 2022;21:524-539 – ISSN 1611-2156
Received: January 18, 2022, accepted: February 15, 2022, published: February 25, 2022

Author, year	Country	Disease	Participants				Modes of exercise	Intervention group
			EX (CON)	BMI	Age	Gender		
								the anaerobic threshold, 21 weeks<br><br>**RT group:**<br>3 sessions/ week; 60-90 min in three training periods: with light loads (40–60 % of 1RM, 3 sets × 15–30 reps; with 60–80 % of 1RM (3 sets × 6–12 reps; higher loads (70–90 % of 1RM, 3 sets × 5–8 reps loads, 21 weeks
Poutafkand, 2020	Iran	Obese	25 (11)	32.2	49	F	AE, RT	**AE group:**<br>3 sessions/week × 45-75 min at an intensity of 50-80 % HRR; 8 weeks<br>**RT group:**<br>3 sessions/week, 3 sets × 10-15 reps at an intensity of 50-65 % 1RM, 8 weeks
Rezaeimanesh, 2020	Iran	Overweight	24 (12)	26.5	25-35	M	HIIT, MICT	**HIIT group:**<br>3 sessions/week, 4-8 × 30 s running at intensity of over 90 % HRmax followed by 30 s rest between each trial, 8 weeks<br>**MICT group:**<br>3 sessions/week × 40-50 min at an intensity of 60-80 % HRmax; 8 weeks
Safarimosavi, 2021	Iran	Prediabetes	24 (8)	26.9	38.7	M	HIIT+ CET	**HIIT group:**<br>2 sessions/week; 10 × 60 seconds at 90 % VO_2peak, 1: 1 work to recovery at 50 W.<br>**CETFAT and CETAT group:**<br>Performed continuous cycling at Fat_{max} and AT intensities, respectively
Scharhag-Rosenberger, 2014	Germany	No disease	37 (37)	25	48.5	Both	RT	3 sessions/week, 2 sets × 16-20 reps at 64-71 % 1RM with 1 min of rest between sets; 6 months
Tofighi, 2017	Iran	Obese	10 (10)	30.3	30.15	F	HIIT	3 sessions/week, 3-8 × 4min intervals at intensity of 90 % target heart rate followed by 2 min recovery between each trial with 50-60 % target heart rate; 8 weeks
Zhao 2017	China	No disease	10 (7)	26	62.1	M	RT	2 times/week × 55 min; 12 weeks

Note: The control group received no training
Abbreviations: MS, metabolic syndrome; HCI, high-intensity concurrent interval; MCC, moderate-intensity continuous concurrent; EE, endurance exercise; SIT, sprint-interval training; AE, aerobic; RT, resistance training; CT, Combined training; CET, continuous endurance training; CETFAT, continuous endurance training with intensity equivalent to fatmax; CETAT, continuous endurance training with intensity equivalent to anaerobic threshold; HICT, high-intensity circuit training; HIIT: high-intensity interval training; MIIT: moderate-intensity training; T2D, type 2 diabetes; HRM, maximum heart rate; HRR, heart rate reserve; 1RM, 1 repetition maximum

EXCLI Journal 2022;21:524-539 – ISSN 1611-2156
Received: January 18, 2022, accepted: February 15, 2022, published: February 25, 2022

Supplementary Table 2: Pooled estimates of irisin within different subgroups

Moderators	No of arms	MD (95 % CI)	P-value	I^2
**Mode of exercise training**				
Aerobic training	12	-0.01 (-0.03, 0.02)	0.60	70 %
Resistance training	22	0.01 (0.00, 0.02)	**0.04**	79 %
Combined	6	0.00 (0.00, 0.01)	**0.002**	0.0 %
**BMI classification**				
< 25 kg/m^2	3	-0.00 (-0.03, 0.03)	0.96	85 %
25-29.9 kg/m^2	29	0.01 (-0.00, 0.01)	0.06	75 %
≥ 30 kg/m^2	5	0.07 (-0.04, 0.18)	0.19	84 %
**Gender**				
Men	6	0.01 (-0.02, 0.03)	0.64	71 %
Women	15	0.01 (-0.00, 0.02)	0.07	49 %
Both	18	0.00 (-0.00, 0.01)	0.19	84 %
**Health status**				
Type 2 diabetes	6	0.00 (0.00, 0.01)	**0.002**	0.0 %
Metabolic syndrome	6	0.45 (-0.08, 0.99)	0.10	0.0 %
Prediabetes	3	0.06 (-0.10, -0.02)	**0.002**	40 %
Multiple sclerosis	1	0.01 (-0.01, 0.02)	0.45	-

Bold value depict statistical significance ($p < 0.05$). MD, mean difference; CI, confidence interval; BMI, body mass index

EXCLI Journal 2022;21:524-539 – ISSN 1611-2156
Received: January 18, 2022, accepted: February 15, 2022, published: February 25, 2022

Supplementary Table 3: Study quality assessment of included studies by the tool for the assessment of study quality in exercise (TESTEX)

**Study Random**	**Eligibility criteria specified**	**Randomization specified**	**Allocation concealment**	**Group similar at baseline**	**Blinding of assessor**	**Outcome measures assessed in 85 % of patients #**	**Intention-to-treat analysis**	**Between-group statistical comparisons were reported***	**Point measures and measures of variability for all reported outcome measures**	**Activity monitoring in control group**	**Relative exercise intensity remained constant**	**Exercise volume and energy expenditure**	**Overall TESTEX (/15)**
Amanat, 2020	1	1	0	1	1	1	0	2	1	0	1	1	10
Azimi Rashti, 2019	1	0	0	1	0	2	0	2	1	0	1	1	9
Bagheri, 2020	1	1	1	1	1	2	0	2	1	0	1	1	12
Banitalebi, 2019	1	1	1	1	1	2	0	2	1	0	1	1	12
Bonfante, 2017	1	0	0	1	0	2	0	2	1	0	1	1	9
Briken, 2016	1	1	0	1	0	1	0	2	1	0	1	1	9
Dianatinasab, 2020	1	0	0	1	1	2	0	2	1	0	1	1	10
Enteshary, 2019	1	0	0	1	0	2	0	2	1	0	1	1	9
Ghanbari-Niaki, 2018	1	0	1	1	0	1	0	2	1	0	0	1	8
Jafari, 2019	1	0	0	1	0	1	0	2	1	0	0	1	7
Jaffari, 2020	1	0	0	1	0	2	0	2	1	0	0	1	8
Kim, 2016	1	0	0	1	0	2	1	2	1	0	1	1	10
Korkmaz, 2019	1	1	0	1	0	2	0	2	1	0	1	1	10
Miyamoto-Mikami, 2015	1	0	0	1	0	1	0	2	1	0	1	1	8
Motahari Rad, 2020	1	1	1	1	1	1	0	2	1	0	1	1	11
Murawska-Cialowicz, 2020	1	0	0	1	0	1	0	2	1	0	1	1	8
Nazari, 2017	1	0	0	1	0	1	0	2	1	0	1	1	8
Pekkala, 2013	1	1	0	1	0	1	0	2	1	0	1	1	9
Poutafkand, 2020	1	0	0	1	0	1	0	2	1	0	0	1	7
Rezaeimanesh, 2020	1	0	0	1	0	1	0	2	1	0	0	1	7
Safarimosavi, 2021	1	0	0	1	0	2	0	2	1	0	1	1	9
Scharhag-Rosenberger, 2014	1	1	0	1	0	2	0	2	1	0	1	1	10
Tofighi, 2017	1	0	0	1	0	1	0	2	1	0	1	1	8
Zhao, 2017	1	1	0	1	0	2	0	2	1	0	0	1	9

Total out of 15 points.
# Three points possible – 1 point if adherence >85 %, 1 point if adverse events reported, 1 point if exercise attendance is reported.
* Two points possible – 1 point if primary outcome is reported, 1 point if all other outcome reported.

EXCLI Journal 2022;21:524-539 – ISSN 1611-2156
Received: January 18, 2022, accepted: February 15, 2022, published: February 25, 2022

Egger Plots

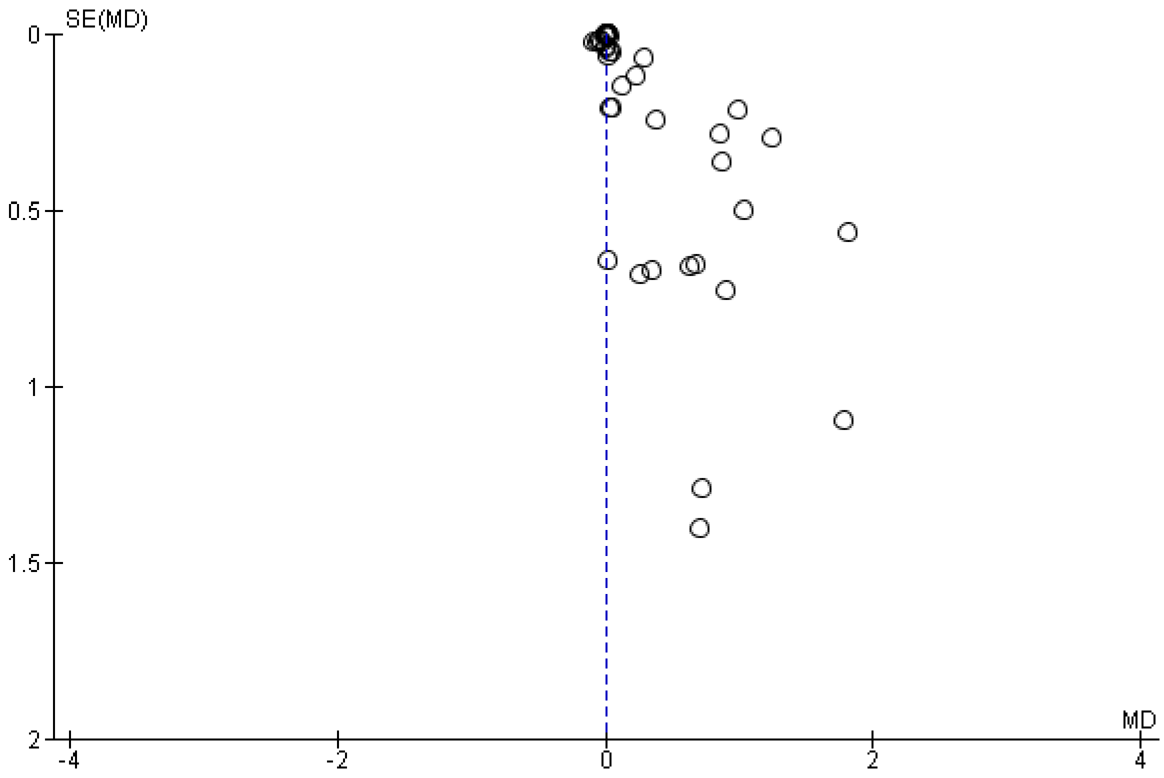

Supplementary Figure 1: Egger plot: irisin

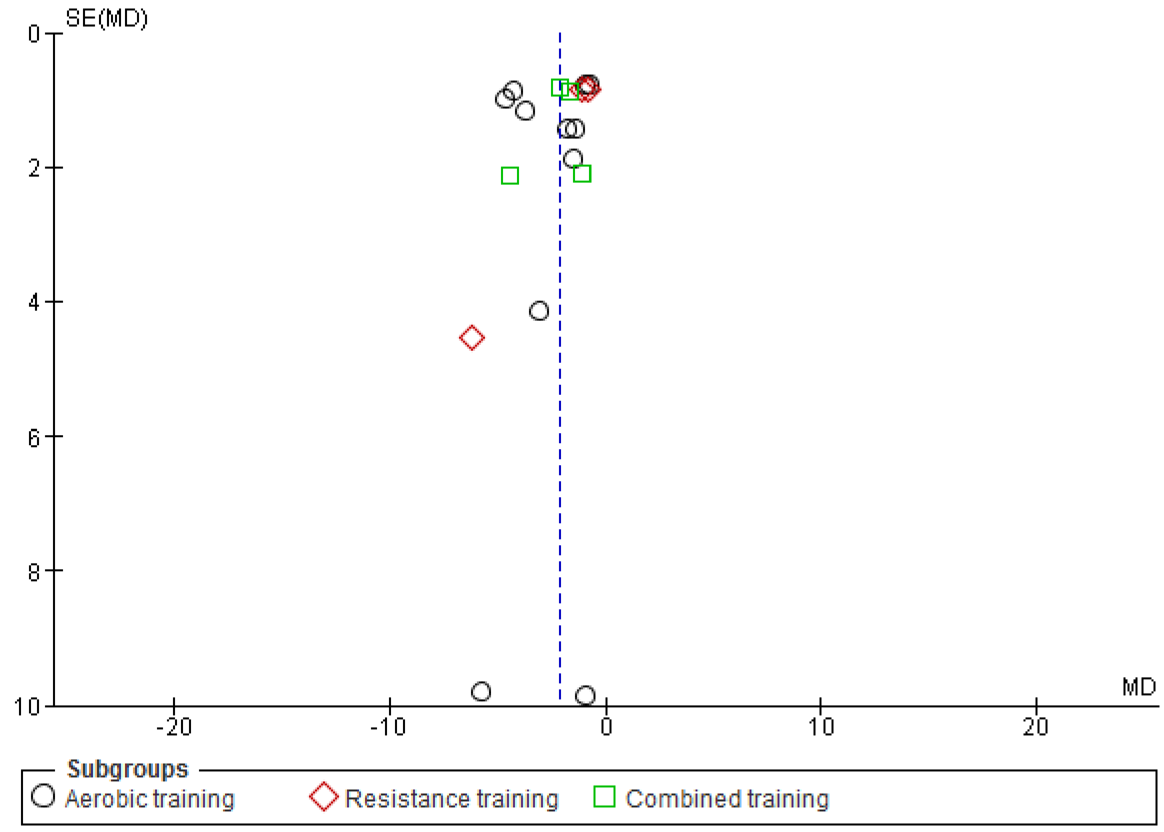

Supplementary Figure 2: Egger plot: insulin

EXCLI Journal 2022;21:524-539 – ISSN 1611-2156
Received: January 18, 2022, accepted: February 15, 2022, published: February 25, 2022

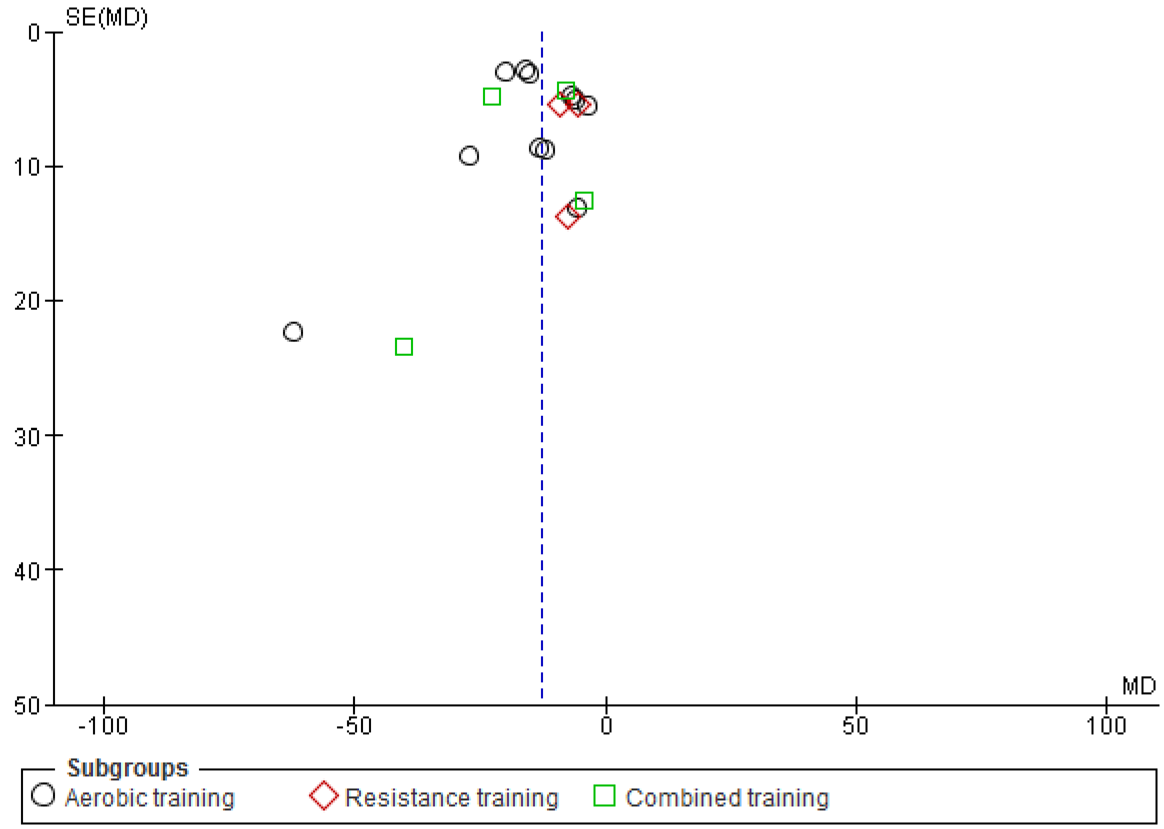

Supplementary Figure 3: Egger plot: glucose

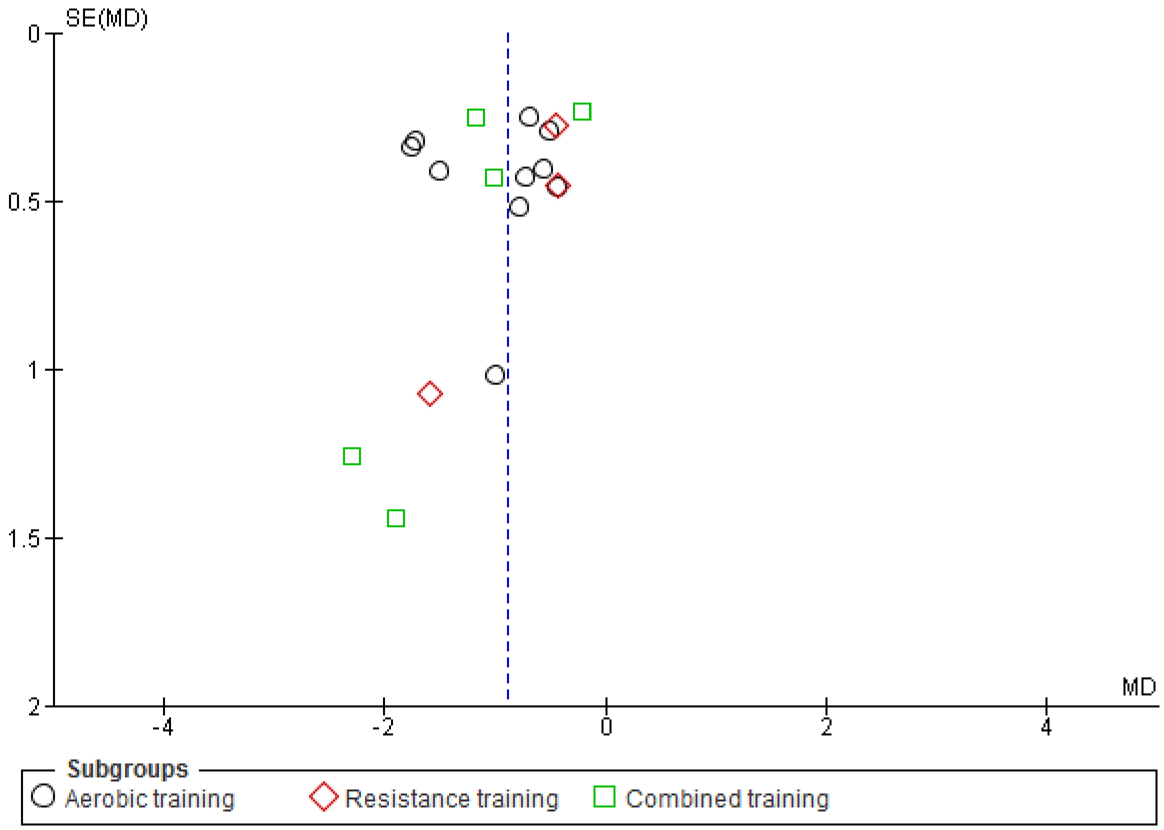

Supplementary Figure 4: Egger plot: HOMA-IR

EXCLI Journal 2022;21:524-539 – ISSN 1611-2156
Received: January 18, 2022, accepted: February 15, 2022, published: February 25, 2022

PubMed Search Strategy

Pub Med Search Strategy Terms

#5 Search """FNDC5""[All Fields] AND (""exercise""[MeSH Terms] OR ""exercise""[All Fields] OR (""exercise""[All Fields] AND ""training""[All Fields]) OR ""exercise training""[All Fields])"
#4 Search "(""irisin""[All Fields] OR ""irisin s""[All Fields]) AND (""exercise""[MeSH Terms] OR ""exercise""[All Fields] OR (""physical""[All Fields] AND ""activity""[All Fields]) OR ""physical activity""[All Fields])"
#3 Search "(""irisin""[All Fields] OR ""irisin s""[All Fields]) AND (""exercise""[MeSH Terms] OR ""exercise""[All Fields] OR (""physical""[All Fields] AND ""activity""[All Fields]) OR ""physical activity""[All Fields])"
#2 Search "(""irisin""[All Fields] OR ""irisin s""[All Fields]) AND (""exercise""[MeSH Terms] OR ""exercise""[All Fields] OR ""exercises""[All Fields] OR ""exercise therapy""[MeSH Terms] OR (""exercise""[All Fields] AND ""therapy""[All Fields]) OR ""exercise therapy""[All Fields] OR ""exercise s""[All Fields] OR ""exercised""[All Fields] OR ""exerciser""[All Fields] OR ""exercisers""[All Fields] OR ""exercising""[All Fields])"
#1 Search """irisin""[All Fields] OR ""irisin's""[All Fields]"
